# Microbial influences on immune modulation and colorectal cancer progression through combined transcriptomic and microbiomic analysis

**DOI:** 10.1002/imo2.70072

**Published:** 2026-01-04

**Authors:** Shuting Xia, Likun Wang, Yangzhuo Xu, Jiaqi Zhang, Zeyang Zhou, Libing Sun, Heng Li, Chungen Xing, Jialu Mao, Yina Liu, Fulin Yang, Ling Zhong, Jiancheng Xu, Xiaodong Yang, Zhemin Zhou

**Affiliations:** ^1^ Department of Clinical Laboratory, The Second Affiliated Hospital of Soochow University, Cancer Institute, Suzhou Medical College Soochow University Suzhou China; ^2^ National Center of Technology Innovation for Biopharmaceuticals Suzhou Biomedical Industry Innovation Center Suzhou China; ^3^ Department of Public Health, School of Public Health, Suzhou Medical College Soochow University Suzhou China; ^4^ Department of General Surgery The Second Affiliated Hospital of Soochow University Suzhou China; ^5^ Department of Pathology, The Affiliated Suzhou Hospital of Nanjing Medical University, Suzhou Municipal Hospital, Gusu School Nanjing Medical University Suzhou China; ^6^ Key Laboratory of Alkene‐carbon Fibres‐based Technology & Application for Detection of Major Infectious Diseases, MOE Key Laboratory of Geriatric Diseases and Immunology, Suzhou Medical College Soochow University Suzhou China; ^7^ Suzhou Key Laboratory of Pathogen Bioscience and Anti‐infective Medicine, Jiangsu Province Engineering Research Center of Precision Diagnostics and Therapeutics Development, Suzhou Medical College Soochow University Suzhou China

## Abstract

Schematic representation of the distinct tumor microenvironment characteristics in CA1 and CA2 subtypes. CA1 illustrates an immune‐active tumor microenvironment characterized by elevated infiltration of immune cells, particularly CD8 + T cells and macrophages, and enhanced IFN‐γ signaling. This subtype shows enrichment of specific bacterial species (including *P. gingivalis* and *B. fragilis*) that contribute to immunomodulation through metabolic pathways such as γ‐aminobutyric acid (GABA) and butanoate metabolism. Despite high immune activity, CA1 exhibits a poor prognosis, potentially linked to immune exhaustion, manifested by high expression of PD‐1, CTLA‐4, LAG‐3, and TIM‐3. CA2 depicts an immune‐suppressed microenvironment featuring reduced immune cell infiltration, lower inflammatory signatures, and a distinct microbial composition dominated by health‐associated bacteria. This tumor phenotype shows better overall survival even with reduced immune activity. Arrows indicate key molecular interactions, with solid lines representing direct effects and dashed lines indicating indirect modulation.

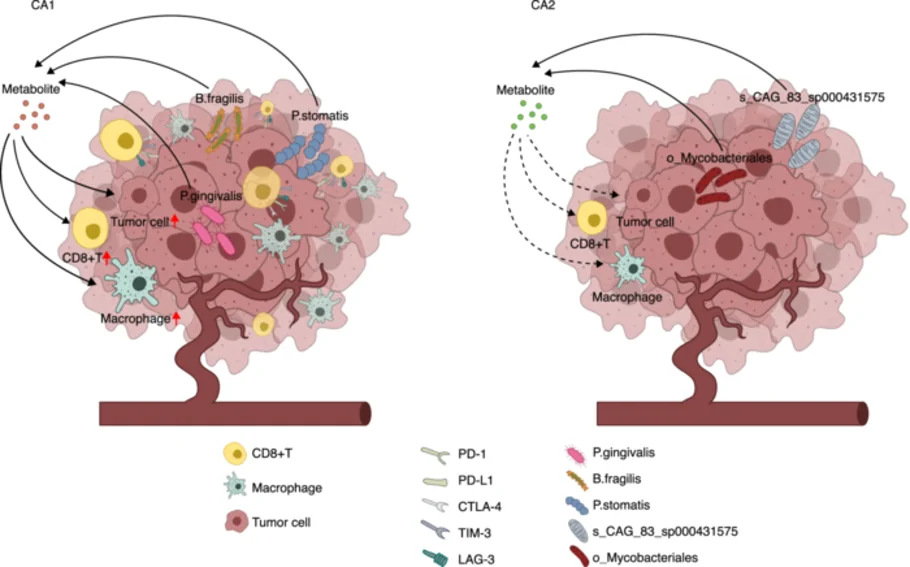

To the Editor,

Colorectal cancer (CRC) represents a significant global health burden, ranking as the third most common cancer and a leading cause of cancer‐related mortality worldwide [1]. Its progression is influenced by a combination of genetic mutations, environmental factors, and immune system interactions. Recent advances have highlighted the in situ microbiome as a crucial yet underexplored factor in CRC pathogenesis [2, 3]. Dysbiosis, or microbial imbalance, contributes to CRC through diverse mechanisms, including chronic inflammation, immune modulation, and metabolic alterations. Despite these findings, the complex interactions between the microbiome and the host immune system, particularly in the context of tumor progression and immune evasion, remain poorly understood. This gap underscores the need for integrative studies that explore these interactions and their potential therapeutic implications.

The immune microenvironment plays a pivotal role in CRC prognosis and response to treatment, yet its considerable heterogeneity complicates patient stratification and clinical treatments. Although frameworks such as the Consensus Molecular Subtypes (CMS) [4] and global immune subtypes provide valuable insights into CRC biology [5], their clinical translation remains limited [6], especially for microsatellite‐stable (MSS) tumors. MSS tumors, which represent the majority of CRC cases, are particularly resistant to immunotherapy, highlighting a critical area of unmet need in treatment [7]. Current approaches also fail to incorporate microbiome‐mediated influences on immune pathways, representing a significant gap in our understanding of CRC's molecular landscape.

Emerging evidence suggests that specific microbes not only contribute to tumor initiation but also shape the immune landscape within the tumor microenvironment [8]. Dysbiosis, the disruption of microbial balance, plays a key role in CRC development through various mechanisms [9]. Pathogenic bacteria, such as *Peptostreptococcus stomatis* [10] and *Peptostreptococcus anaerobius* [11], have been implicated in promoting immune evasion and tumor progression. Conversely, certain microbes may enhance anti‐tumor immunity, suggesting a dual role for the microbiome in modulating CRC outcomes [12]. However, most studies focus on either microbial or immune factors in isolation, overlooking the complex interplay between these systems.

Technological advances in sequencing, particularly the 2b‐RAD‐M technique [13], now enable species‐level resolution even in samples with high host contamination. In this study, we employed an integrated analysis of transcriptomic and in situ microbiomic data from paired tumor and adjacent normal tissues in 31 CRC patients. Our findings reveal the presence of distinct immune‐microbial subtypes, CA1 and CA2, characterized by unique immune activities and microbial compositions. Despite heightened immune infiltration and activation of pro‐inflammatory pathways in CA1, this subtype is associated with a poor prognosis, likely due to immune exhaustion characterized by high expression of checkpoint molecules and the enrichment of *Porphyromonas gingivalis* and *Bacteroides fragilis*. These results highlight the crucial role of specific microbes in shaping the immune microenvironment of CRC and suggest that microbiome‐targeted therapies may hold promise for improving the efficacy of immunotherapy.

## 1 TRANSCRIPTOMIC AND MICROBIOMIC SIGNATURES REVEAL POTENTIAL MOLECULAR SUBTYPES OF CRC

Transcriptomic profiling of 31 CRC pairs (CA: tumor, PCA: adjacent normal tissues; Table S1) showed a 3272‐gene tumor‐specific expression signature enriched for IL‐17‐driven immunity and proliferation/metabolism networks (Figure S1A–C), while 2b‐RAD‐M microbiome data revealed alpha‐diversity was reduced in tumors, but beta‐diversity remained stable (Figure S1D,F). Despite pronounced tumor‐versus‐normal transcriptomic divergence, within‐patient microbial communities were more similar, with most compositional variance attributable to inter‐individual rather than tissue‐specific differences (Figure 1A,B). This discordance supports a host‐centric stratification framework in CRC.

## 2 SUBTYPE CLASSIFICATION BASED ON IMMUNE AND MICROBIAL SIGNATURES

We integrated microbial functional genes (Kyoto Encyclopedia of Genes and Genomes orthologs, KOs) with host gene expression data to construct a heterogeneous co‐expression network. Using Kendall's correlation, we identified 2454 microbial KOs in tumor samples significantly associated with 3196 host genes involved in various biological processes. These interactions assembled 206 tumor‐specific microbial–host gene modules (Figure 1C); compared with the 15 co‐expression clusters produced by Weighted Gene Co‐expression Network Analysis (WGCNA) alone (Figure S2), integration of transcriptomic–microbiomic achieves far finer resolution, compresses within‐group heterogeneity, and generates biologically sharper strata.

**Figure 1:**
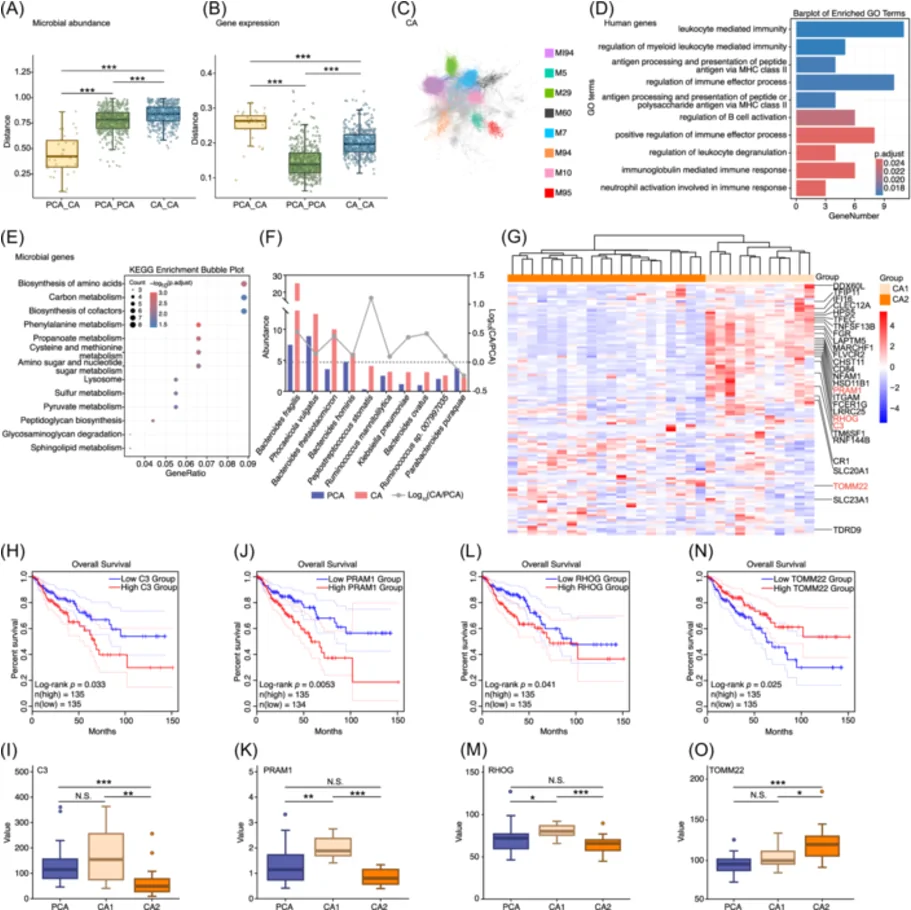
Subtype classification based on integrated analysis of transcriptome and microbiome. (A) Box plots for Bray‐Curtis distances of microbial profiles between sample pairs comprising CA (tumor) and PCA (adjacent normal tissue) samples from the same individuals (PCA_CA), or between PCA and CA samples from different patients. Samples from the same patients exhibited the lowest distances. (B) Box plots for Bray‐Curtis distances of gene transcriptions between sample pairs that comprising CA and PCA samples from the same individuals (PCA_CA), or between PCA and CA samples from different patients. (C) Graph of host‐microbe interactions in colorectal cancer samples. Nodes represent host gene modules and microbial species, with edges indicating significant correlations (*p* < 0.01, *q* < 0.2, |*τ* | ≥ 0.3). Edge thickness represents correlation strength. The graph showed the top 8 modules with the most nodes among the total 206 modules, with Module 95 (M95) highlighted as a central hub connecting immune‐related processes with microbial interactions. (D) Gene Ontology (GO) enrichment analysis of host genes within significant modules. Top 10 enriched biological processes are shown, with bar length representing the numbers of associated genes and color intensity indicating enrichment significance. (E) Kyoto Encyclopedia of Genes and Genomes (KEGG) pathway enrichment analysis of bacterial genes in M95. The bubble plot shows significantly enriched pathways (adjusted *p* < 0.05), with dot size indicating gene count and color representing enrichment significance. (F) Relative contribution of bacterial species to the enriched KEGG pathways. A stacked bar plot shows the contribution of the top 10 species to the enriched genes. The overlay grey curve shows the relative ratio of each species contributing to the CA and PCA samples. (G) Unsupervised hierarchical clustering of CRC samples based on M95 module gene expression patterns identifies two distinct subtypes (CA1 and CA2). Heatmap shows normalized expression values of M95 genes (rows) across samples (columns). Color scale represents *z*‐scored expression levels. (Black genes are 24 genes derived from random forest, red genes are four prognostic genes.) (H, J, L, N) Kaplan–Meier survival analyses for four prognostic genes in CRC patients. High expression (red) correlates with worse overall survival compared to low expression (blue) for genes of C3 (H), PRAM1 (J), and RHOG (L). TOMM22 (N) exhibited the opposite trend. *p* values determined by the log‐rank test. (I, K, M, O) Expression levels of the four prognostic genes in CA1 versus CA2 subtypes: Box plots show significantly higher expression in CA1 samples, consistent with poorer clinical outcomes. N.S. not significant, *p < 0.05, **p < 0.01, ***p < 0.001 by one‐way ANOVA. ANOVA, analysis of variance.

Most of the 206 tumor‐specific host–microbe modules were enriched for host genes associated with cell cycle regulation and DNA replication (Figure S3), but M95 stood out as an immune‐centric network: 109 genes driving leukocyte trafficking, MHC‐II antigen presentation, and immune cell activation were selectively upregulated in tumors (Figure 1D and Table S2). Its enriched microbial KOs (phenylalanine/propionate metabolism, peptidoglycan synthesis) (Figure 1E) trace predominantly to *B. fragilis* and *P. stomatis* (Figure 1F), implicating these species in microbiota‐mediated immune modulation within the CRC microenvironment. Compared to the clusters from WGCNA, M95 largely represented a subset of cluster 1, revealing specific associations between immunity and microbes (Figure S2B). We subdivided CRC samples into two subtypes based on host gene expression in the M95 module: CA1 (11 samples) and CA2 (20 samples) (Figure 1G and Figure S4).

## 3 CA1 TUMORS EXHIBIT WORSE SURVIVAL OUTCOMES AND SIGNATURES OF T CELL EXHAUSTION

Four of the 109 M95 genes exhibited significant survival differences between patients with CA1 and CA2 tumors, according to the GEPIA2 database (C3, PRAM1, RHOG, and TOMM22). The gene expression patterns in CA1 tumors were always associated with worse survival outcomes (Figure 1H−O).

Using the ESTIMATE immune scoring method, CA1 tumors exhibited higher immune scores than CA2 tumors (Figure 2A) and higher immune cell infiltration, particularly CD8 + T‐cell infiltration (Figure 2B−D), which was confirmed by immunohistochemical staining (Figure 2E−G). Expression of IFNG and interferon‐stimulated genes (ISGs) was also upregulated in CA1, but Gene Set Variation Analysis (GSVA) scores for the immune checkpoint molecules were significantly higher in CA1 than in CA2 (Figure 2H and Figure S5A–D).

**Figure 2:**
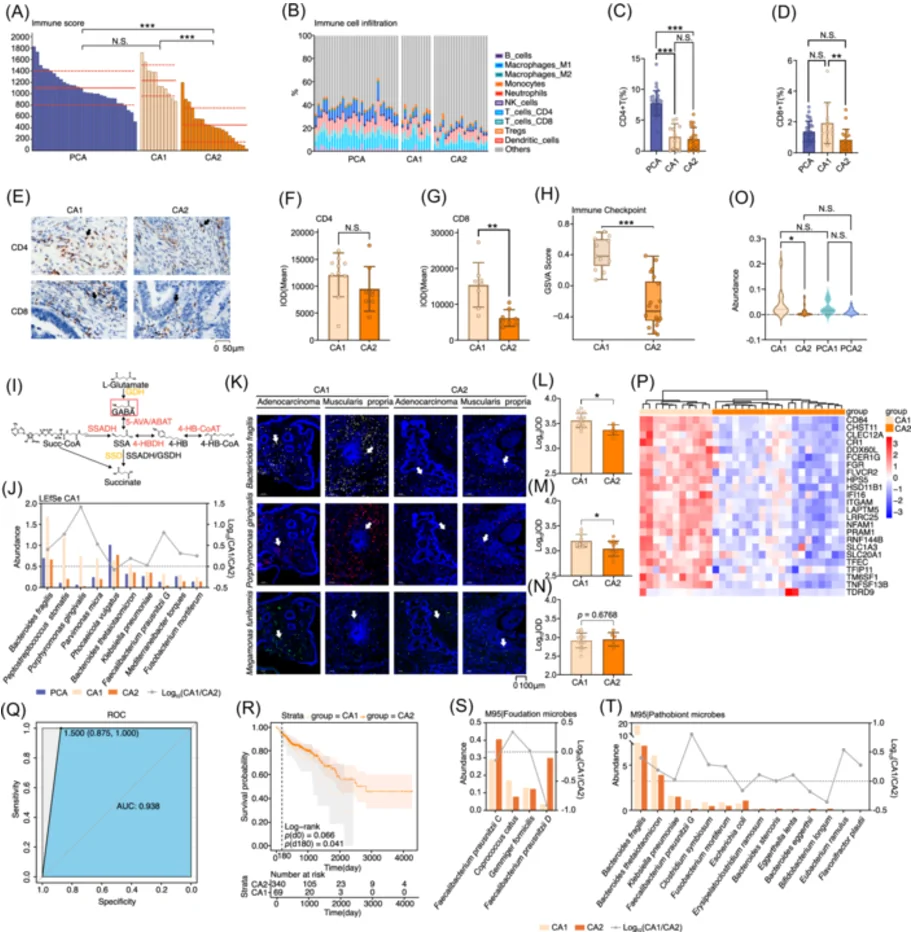
Immune and microbial characterization of subtypes of CRC. (A) Comparison of immune activity scores by CIBERSORT among PCA, CA1, and CA2 subtypes. Each bar represents an individual sample. The solid red horizontal lines indicate the mean value, and the dashed red lines represent the mean ± standard deviation (SD). ***p < 0.001, by one‐way ANOVA. (B) Predicted immune cell infiltration levels across subtypes were quantified using the CIBERSORT algorithm. (C, D) Relative proportions of specific immune cell populations significantly differ between CA1 and CA2 subtypes: Bar plots show mean value and standard deviation; points indicate individual samples. *p < 0.05, **p < 0.01, ***p < 0.001 by one‐way ANOVA. (E–G) Immunohistochemistry (IHC) validation of immune cell populations: (E) Representative IHC images showing CD8 and CD4 staining in CA1 and CA2 samples. Scale bars, 50 µm. (F) Integrated Optical Density (IOD) of CD4 cell markers between CA1 and CA2 samples. (G) IOD of CD8 cell markers between CA1 and CA2 samples. Bar plots show the mean value and standard deviation. **p < 0.01 by Student's *t*‐test. (H) Comparison of Gene Set Variation Analysis (GSVA) scores of checkpoint molecules between CA1 and CA2 subtypes. ***p < 0.001 by Student's *t*‐test. (I) KEGG pathway analysis reveals enrichment of GABA metabolism‐related pathways in CA1‐associated microbiota. Red: >1.5 × enrichment, yellow: >1× enrichment. (Abbreviations: 4‐HB, 4‐hydroxybutyric acid; 4‐HB‐CoA, 4‐hydroxybutyryl‐CoA; 4‐HB‐CoAT, 4‐HB‐CoA transferase; 4‐HBDH, 4‐hydroxybutyric acid dehydrogenase; 5‐AVA/ABAT, 5‐aminovaleric acid/4‐aminobutyrate transaminase; GABA, γ‐aminobutyric acid; GDH, glutamate dehydrogenase; GSDH, succinate dehydrogenase; SSADH, succinate semialdehyde dehydrogenase; SSA, succinate semialdehyde; SSD, succinate semialdehyde dehydrogenase) (J) Relative contribution of bacterial species to enriched KEGG pathways. Bar plot shows the proportion of pathway‐specific genes contributed by each species (top 10 species contributed to CA1) in PCA (blue), CA1 (light‐orange), or CA2 (orange) samples. The dotted curve shows the relative contributions of species in CA1 versus CA2. (K) Fluorescence in situ hybridization (FISH) analysis of FFPE tissue samples using species‐specific probes. Representative images showing bacterial colonization patterns in CA1 and CA2 tissues. Scale bars, 100 µm. Arrows show examples of detected bacterial cells. (L−N) Quantification of FISH signals for three key bacterial species: (L) *Bacteroides fragilis*; (M) *Porphyromonas gingivalis*; (N) *Megamonas funiformis*. Bar plots show bacterial abundance scores across CA subtypes. *p < 0.05 by Student's *t*‐test. (O) Violin plots displaying the differential relative bacterial abundance in paired CA and PCA samples. *p < 0.05 by one‐way ANOVA. (P) The heatmap shows biomarkers derived from a Random forest model distinguishing CA1 and CA2 subtypes. (Q) Receiver operating characteristic (ROC) curve of the Random Forest model. The area under the curve (AUC) is 0.938. (R) Kaplan–Meier survival analysis comparing overall survival between CA1‐like and CA2‐like patients in the TCGA cohort. CA1‐like patients (light orange) show significantly worse survival compared to CA2‐like patients (orange) after the initial 180 days (*p* = 0.04). *p* Values determined by the log‐rank test. Hazard ratios (HR) with 95% confidence intervals are shown. (S, T) Relative abundance of Foundation and Pathobiont microbes (health‐ or disease‐associated microbes described in [14]) in CA1 versus CA2 samples. Grey curves show relative abundances between the two groups.

The immune activity observed in CA1 tumors highlights the complex nature of tumor–immune interactions. While immune pathways related to IFNG and ISGs are upregulated in CA1, generally associated with effective anti‐tumor immunity, sustained stimulation of IFNG will induce tumor immune evasion by promoting the expression of immune checkpoint molecules, such as PD‐L1, PD‐1, CTLA‐4, LAG‐3, and TIM‐3, which were also found to be increased in the CA1 subset (Figure S5C). Persistent high expression of immune checkpoint molecules typically marks immune exhaustion [15], indicating that cytotoxic T‐cell effector function is compromised. These findings provide a plausible explanation for the poorer prognosis in this subtype.

## 4 MICROBIAL ENRICHMENT IN CA1: THE ROLE OF *P. GINGIVALIS* AND *P. STOMATIS*

Microbial profiling of the tumor tissues revealed that tumors were enriched with *B. fragilis* and *P. stomatis* (Figure 1F), which have been previously implicated in CRC. Further analysis using LDA effect size (LEfSe) and Wilcoxon rank‐sum tests demonstrated that *P. gingivalis* and *Megamonas funiformis* were enriched in CA1 tumors, while *Mycobacteriales* and *CAG_83_sp000431575* were more prevalent in CA2 tumors (Figure S6). These findings suggest that specific bacteria contribute to the immune and metabolic features of the CA1 subtype.

To explore the functional implications of this microbial composition, we analyzed the microbial gene pathways using KOs. CA1 tumors exhibited distinct enrichment of KOs associated with metabolic pathways, particularly butanoate metabolism (Figure S7A–C). Interestingly, genes involved in γ‐aminobutyric acid (GABA) metabolism were significantly upregulated in CA1 tumors (Figure 2I). GABA has been frequently reported to be associated with the promotion of tumor cell proliferation and the regulation of the tumor immune microenvironment. For example, GABA secreted by B cells mediates the immunosuppressive state in CRC [16] and those induced by sleep deprivation promote the proliferation and migration of colon tumor cells [17]. These findings suggested that the alteration of short‐chain fatty acids (SCFA) metabolic pathways might have contributed to the cancer‐microbial interactions.

Notably, *B. fragilis*, *P. stomatis*, and *P. gingivalis* were key microbial drivers of these enriched pathways, with *P. gingivalis* and *P. stomatis* showing a 25‐fold and sixfold higher abundance in CA1 tumors compared to CA2, respectively (Figure 2J). These bacteria also significantly contributed to KOs associated with the M95 and IL‐17 signaling pathway (Figure S8A,B). Functional enrichment analysis of upregulated genes in the microbial enrichment group revealed a strong association with immune‐related pathways, further implicating them in immune modulation (Figure S9A–C).

It is particularly worth mentioning that *P. stomatis* and *P. gingivalis* are both typical oral bacteria, and the enrichment of oral bacteria is associated with the depletion of the gut microbiota and patient outcomes [18]. The persistent presence of these microbes in CA1 tumors likely contributes to immune suppression and poor prognosis, making them potential targets for microbiome‐based therapies.

To validate our microbiome findings, we utilized fluorescence in situ hybridization (FISH) to detect the presence of *B. fragilis*, *M. funiformis*, and *P. gingivalis* in tumor tissues from the same set of CRC patients. As illustrated in Figure 2K−N, *B. fragilis* and *P. gingivalis* were significantly enriched in CA1 tumors compared to CA2 tumors, whereas *M. funiformis* did not exhibit a significant differential abundance. These results confirm the preferential colonization of specific bacteria in CA1 tumors. Compared with adjacent normal tissue, three specific bacteria (*B. fragilis*, *P. stomatis*, and *P. gingivalis*) exhibit cancer tissue‐specific enrichment (Figure 2O), even though the overall differences tend toward systemic variations (Figure 1A).

## 5 ESTABLISHMENT OF THE RANDOM FOREST MODEL AND MULTI‐COHORT VERIFICATION

To identify a minimal gene set for reliable classification of CRC tumors into CA1 and CA2 subtypes, we applied a random forest model using 109 genes from the M95 module. Twenty‐four genes sufficed to classify subtypes with an Area Under the Curve (AUC) of 0.938. (Figure 2P,Q and Table S3).

Using the 24 genes generated from the random forest model, we further refined the classification and successfully separated CRC samples in The Cancer Genome Atlas (TCGA) cohort into CA1 and CA2 subtypes (Figure S10A). Compared with previously described immunity types [5] using their established classification tools, we found that the majority of CA2 tumors were predominantly assigned to the c1 subtype, which is characterized as the “wound‐healing” type, while over half of the CA1 tumors were classified as subtype c2, associated with an IFN‐γ dominant immune response (Figure S10B). Principal coordinate analysis (PCoA) further confirmed the association, with CA2 and c1 samples, and CA1 and c2 samples grouped together, respectively (Figure S10C).

Interestingly, no survival differences were observed between immunity subtypes c1 and c2 in the TCGA dataset, suggesting that these immune classifications alone may not fully explain the observed differences in clinical outcomes. Starting from Day 180, there was a statistically significant survival difference between the two subtypes (Figure 2R), indicating that the CA1 subtype is associated with poorer long‐term prognosis. These results highlight the utility of our classification system, which provides a more precise way to categorize CRC tumors based on microbial and immune signatures. This approach could potentially inform personalized treatment strategies and help predict patient outcomes.

Unlike CMS, which assigns subtypes primarily according to cancer‐cell‐intrinsic pathways, our classification focuses on microbial‐related host‐immune genes and thus re‐stratifies tumors purely from an immunological perspective. We also evaluated all our samples with CMScaller, which assigned each sample to one of four CMS types. The results exhibited low associations with our subtypes (Figure S10D). This is not particularly surprising, because early studies have also revealed low correlations between the immunity types and the CMS types [19].

In line with our findings, a recent study has identified two antagonistic sets of gut microbiomes, one associated with healthy status and the other with disease [14]. Our analysis showed that CA2 tumors were enriched with “healthy” foundational microbiota, while CA1 tumors were dominated by pathobiont microbes (Figure 2S,T). This shift in microbial composition, where beneficial bacteria are displaced by pathogenic microbes, is consistent with the concept of microbial dysbiosis, which has been shown to contribute to CRC progression and poor prognosis.

In summary, we identified two immune subtypes CA1 and CA2 of CRC by their different immune landscape. Especially, CA1 is characterized by increased immune infiltration, elevated IFN‐γ and checkpoint molecular expression, specific microbial aggregation, and poor prognosis, whereas CA2 showed lower immune infiltration and better prognosis. Further exploration demonstrated that specific microbes such as *B. fragilis* and *P. gingivalis* may stimulate the CRC immune microenvironment through metabolites such as GABA. We speculate that the presence of these specific microbes could be a key factor shaping the immune state of tumors in CRC, potentially enhancing the efficacy of immune checkpoint inhibitors. Gene sets and microbes could serve as biomarkers for identifying different types of CRC.

## AUTHOR CONTRIBUTIONS

**Shuting Xia**: Methodology; visualization; writing—original draft; writing—review and editing. **Likun Wang**: Data curation; formal analysis; visualization; writing—original draft. **Yangzhuo Xu**: Data curation; validation and visualization. **Jiaqi Zhang**: Resources; data curation. **Zeyang Zhou**: Data curation; formal analysis. **Libing Sun**: Resources; project administration. **Jialu Mao**: Resources; project administration. **Yina Liu**: Resources; project administration. **Heng Li**: Investigation; data curation; writing—review and editing. **Chungen Xing**: Resources; data curation; project administration. **Fulin Yang**: Validation; data curation; software. **Ling Zhong**: Formal analysis; visualization. **Jiancheng Xu**: Resources; supervision; funding acquisition. **Xiaodong Yang**: Resources; investigation; funding acquisition. **Zhemin Zhou**: Conceptualization; supervision; funding acquisition. All authors have read and agreed to the published version of the manuscript.

## CONFLICT OF INTEREST STATEMENT

The authors declare no conflicts of interest.

## ETHICS STATEMENT

The ethics application (JD‐LK‐2020‐004‐01) was approved by the Research Ethics Committee of The Second Affiliated Hospital of Soochow University.

## Supporting information


**Figure S1.** Integrated analysis of transcriptome and microbiome profiles in colorectal cancer.
**Figure S2.** Comparison between clusters from transcriptome and modules from microbiome‐transcriptome network.
**Figure S3‐1.** The microbial KO pathway is enriched in each module of the CA group.
**Figure S3‐2.** The host gene pathway is enriched in each module of the CA group.
**Figure S4.** M95‐based molecular classification.
**Figure S5.** Immune signatures of subtypes.
**Figure S6.** Microbial enrichment of subtypes.
**Figure S7.** Differential microbial KO analysis of CA1 and CA2 groups.
**Figure S8.** Microorganisms screened from KOs of M95 and IL‐17 are similar to CA1.
**Figure S9.** GSEA gene expression difference analysis.
**Figure S10.** Clinical validation of CA subtypes.

**Table S1.** Clinical information of patients.
**Table S2.** 109 genes of M95.
**Table S3.** 24 genes selected by random forest.

## Data Availability

All the sequencing data have been deposited in the NCBI BioProject database under accession number PRJNA1209391 (https://www.ncbi.nlm.nih.gov/bioproject/PRJNA1209391). The data and code used to generate the main figure have been deposited on GitHub and are publicly accessible at https://github.com/YangzhuoXu/crc_data. Supplementary materials (methods, figures, tables, graphical abstract, slides, videos, Chinese translated version and update materials) may be found in the online DOI or iMetaOmics http://www.imeta.science/imetaomics/.
