# Supplementary material for: Microbial influences on immune modulation and colorectal cancer progression through combined transcriptomic and microbiomic analysis
Source: IMetaOmics. 2026 Jan 4;3(1):e70072. doi: 10.1002/imo2.70072 (PMC13674408; doi:10.1002/imo2.70072)
Supplement: Supplementary file 1 [file IMO2-3-e70072-s002.docx]

Supporting information to

**Microbial Influences on Immune Modulation and Colorectal Cancer Progression Through Combined Transcriptomic and Microbiomic Analysis**

**Running title**: **Microbiome-Immune Crosstalk in Colorectal Cancer**

Shuting Xia^1,2#^, Likun Wang^3#^, Yangzhuo Xu^3#^, Jiaqi Zhang^1#^, Zeyang Zhou^4#^, Libing Sun^5#^, Heng Li^1^, Chungen Xing^4^, Jialu Mao^4^, Yina Liu^1^, Fulin Yang^1^, Ling Zhong^1^, Jiancheng Xu^1,2*^, Xiaodong Yang^1,2,4*^, Zhemin Zhou^1,2,6,7*^

^1^The Second Affiliated Hospital of Soochow University, Cancer Institute, Suzhou Medical College, Soochow University, Suzhou, 215127, China

^2^National Center of Technology Innovation for Biopharmaceuticals, Suzhou Biomedical Industry Innovation Center, Suzhou, 215127, China

^3^School of Public Health, Suzhou Medical College, Soochow University, Suzhou, 215127, China

^4^Department of General Surgery, The Second Affiliated Hospital of Soochow University, Suzhou, 215004, China.

^5^The Affiliated Suzhou Hospital of Nanjing Medical University, Suzhou Municipal Hospital, Gusu School, Nanjing Medical University, Suzhou, 215002, China

^6^Key Laboratory of Alkene-carbon Fibres-based Technology & Application for Detection of Major Infectious Diseases, MOE Key Laboratory of Geriatric Diseases and Immunology, Suzhou Medical College, Soochow University, Suzhou, 215127, China

^7^Suzhou Key Laboratory of Pathogen Bioscience and Anti-infective Medicine, Jiangsu Province Engineering Research Center of Precision Diagnostics and Therapeutics Development, Suzhou Medical College, Soochow University, Suzhou, 215127, China

^#^These authors contributed equally: Shuting Xia, Likun Wang, Yangzhuo Xu, Jiaqi Zhang, Zeyang Zhou, Libing Sun

*Correspondence:

[jcxu@suda.edu.cn](mailto:jcxu@suda.edu.cn) (Jiancheng Xu)

yangxiaodong2366@suda.edu.cn (Xiaodong Yang)

[zmzhou@suda.edu.cn (Zhemin Zhou)](mailto:zmzhou@suda.edu.cn%20(Zhemin%20Zhou))

**LIST OF SUPPLEMENTARY INFORMATION**

**Supplementary Methods**

**Supplementary Figures**

**Figure S1.** Integrated analysis of transcriptome and microbiome profiles in colorectal cancer.

**Figure S2.** Comparison between clusters from transcriptome and modules from microbiome-transcriptome network.

**Figure S3-1.** The microbial KO pathway is enriched in each module of the CA group.

**Figure S3-2.** The host gene pathway is enriched in each module of the CA group.

**Figure S4.** M95-based molecular classification.

**Figure S5.** Immune signatures of subtypes.

**Figure S6.** Microbial enrichment of subtypes.

**Figure S7.** Differential microbial KO analysis of CA1 and CA2 groups.

**Figure S8.** Microorganisms screened from KOs of M95 and IL-17 are similar to CA1.

**Figure S9.** GSEA gene expression difference analysis.

**Figure S10.** Clinical validation of CA subtypes.

**METHODS**

**Study Design and Patient Cohort**

This study was designed as a cross-sectional analysis involving 31 patients diagnosed with colorectal cancer (CRC) who underwent surgical resection at the Second Affiliated Hospital of Soochow University. Inclusion criteria encompassed patients aged 18 years or older, with histologically confirmed CRC, and no prior treatment (chemotherapy, radiotherapy, or immunotherapy) before surgery. All patients provided written informed consent, and the study was approved by the Institutional Review Board of Suzhou Medical College.

**Sample Collection**

Paired tumor (CA) and adjacent normal tissues (PCA) were collected during surgical procedures. Samples were immediately snap-frozen in liquid nitrogen and stored at -80°C until further analysis. The pathological diagnosis was confirmed by a pathologist, ensuring that only tumor tissues with a minimum of 70% tumor cell content were included in the analysis.

**Transcriptomic Analysis**

RNA was extracted from both tumor and adjacent normal tissues using the RNeasy Mini Kit (Qiagen). The quality and quantity of RNA were assessed using a Bioanalyzer (Agilent Technologies) [1]. Libraries for RNA sequencing were prepared using the TruSeq Stranded mRNA Library Prep Kit (Illumina) following the manufacturer's protocols. Sequencing was performed on an Illumina NovaSeq 6000 platform, generating paired-end reads of 150 bp.

Raw sequencing data were processed using FastQC (By Simon Andrews, version 0.12.1) for quality control checks and trimmed using Trimmomatic [2] to remove low-quality bases and adapter sequences. Cleaned reads were aligned to the human genome (GRCh38) using STAR [3] aligner. Differential gene expression analysis was conducted using edgeR [4], with a significance threshold set at FDR < 0.05.

**Microbiomic Analysis**

DNA was extracted from tissue samples using the DNeasy PowerSoil Kit (Qiagen) according to the manufacturer's instructions. Metagenomic sequencing was performed using the 2b-RAD-M technique [5], which employs type IIB restriction endonucleases to generate isoschizomers for microbial community analysis. Library preparation and sequencing were conducted on an Illumina platform with paired-end reads of 150 bp. Sequencing depth and coverage were assessed to ensure adequate representation of microbial diversity.

**Microbial Diversity Analysis**

The R package vegan [6] was used for microbial data diversity analysis, calculating diversity indices. Alpha diversity indices included the Shannon and Simpson indices, while beta diversity was assessed using Principal Coordinate Analysis (PCoA).

**Integration of Transcriptomic and Microbiomic Data**

We constructed a co-expression network integrating microbial functional genes (KEGG orthologs) with host gene expression data. To this end, Kendall correlation (*p* < 0.01, *q* < 0.2, |*τ*| ≥ 0.3) was used to assess associations between microbial KOs and host genes, calculated using the R package psych (By William Revelle, version 2.4.3). Modules were constructed using the Gephi software [7] with a resolution of 1.0. The significance of modules was determined using permutation tests.

**Unsupervised Hierarchical Clustering**

The R package pheatmap (By Raivo Kolde, version 1.0.12) was used for heatmap visualization and parameter processing, employing either complete or average linkage as the unsupervised hierarchical clustering method.

**Enrichment Analysis Methods**

The R package clusterProfiler [8] was used for GO and KEGG enrichment analysis. R package org.Hs.eg.db (By Marc Carlson, version 3.22) provided annotation information for human genes. The Benjamini-Hochberg method was used for *p*-value adjustment, with a *p*-value threshold of 0.05 and a *q*-value threshold of 0.2. GSEA enrichment analysis was conducted using the WebGestalt tool [9] for Gene Set Enrichment Analysis between the PCA and CA groups, with the Functional Database set to KEGG pathway.

**Inference of Immune Infiltration**

The R package IOBR (Immuno-Oncology Biological Research) [10] was applied, integrating eight methods (CIBERSORT, TIMER, xCell, MCPcounter, ESTIMATE, EPIC, IPS, and quanTIseq) to decipher information about the tumor microenvironment. In our analysis, we utilized ESTIMATE and quanTIseq algorithms. We conducted Gene Set Variation Analysis (GSVA) using the R package GSVA [11] to evaluate the enrichment scores of specific gene sets across sample groups.

**Immunohistochemical Analysis**

For immunohistochemical analysis, formalin-fixed paraffin-embedded tissue sections were subjected to antigen retrieval, peroxidase blocking, and incubated with the primary antibody (CD4, CD8) overnight at 4°C. DAB was used for signal detection, followed by hematoxylin counterstaining to visualize cell nuclei. The slides were then dehydrated, cleared, and mounted for microscopic examination, allowing for the identification of specific antigens with brown precipitate against a blue nuclear background.

**Fluorescence *In situ* Hybridization (FISH)**

Tissue sections were first treated in a citrate buffer (pH 6.0) for antigen retrieval at 90°C for 48 minutes, followed by 10 minutes digestion with proteinase K at 40°C. Subsequently, the following probes were used: *Megamonas funiformis* with the sequence GCCTCAGCGTCAGTTACAGTCCAG, *Porphyromonas gingivalis* with the sequence CAATACTCGTATCGCCCGTTATTC, and *Bacteroides fragilis* with the sequence GTTTCCACATCATTCCACTG, all hybridized at a dilution of 1.5:100 and 40°C. Tissues were rinsed with PBS and fixed in an animal-specific in situ hybridization fixative for over 12 hours at 4°C. Subsequently, 3mm-thick tissue blocks were dehydrated through a graded ethanol series, cleared in xylene, and embedded in paraffin. Sections of 4μm thickness were obtained and subjected to a dewaxing protocol involving sequential treatments with two dewaxing solutions, anhydrous ethanol, and graded alcohols, culminating in DEPC water immersion. Tissues were repaired using tissue-specific conditions and digested with protease K (20μg/mL) at 37°C, followed by washing with PBS. Pre-hybridization and hybridization were performed at 37°C, with the latter involving an overnight incubation with probe-containing hybridization solution. Post-hybridization washes were conducted in a series of SSC solutions. Nuclei were counterstained with DAPI and images were visualized under a Nikon upright fluorescence microscope.

**Survival Curve GEPIA2**

Survival data for individual genes were retrieved from GEPIA2 [12] using the COAD dataset, and Overall Survival (OS) was analyzed.

**LEfSe Analysis**

LEfSe [13] analysis was conducted on the Wekemo Bioincloud platform to determine the differential expression of microbial species and KOs between the CA1 and CA2 groups. A standard scaling factor of 1,000,000 and an LDA threshold of 1 were used.

**Prognosis Inference for CA Subgroups**

TCGA COAD data were downloaded from the UCSC Xena database (https://xenabrowser.net/datapages/) to infer prognosis differences between the CA1 and CA2 groups, comparing immune characteristics based on the typing analysis from article [14].

**Random Forest Model**

To construct an optimal classification gene set within the M95 module, we employed the Random Forest algorithm using the R package randomForest (By Andy Liaw and Matthew Wiener, version 4.7-1.1). We performed parameter tuning and model validation using 10-fold cross-validation implemented via the caret package [15]. To ensure reproducibility, the random seed was set to 222.

We systematically optimized the hyperparameters ntree (number of trees) and mtry (number of variables randomly sampled as candidates at each split). Specifically, we evaluated ntree values of 200, 500, 1000, and 1500, and screened mtry values ranging from 1 to 50. The optimal model was identified based on the lowest Out-of-Bag (OOB) error and the highest classification accuracy. The final selected hyperparameters were ntree = 500, mtry = 36, and a nodesize = 3.

This optimized model achieved a predictive accuracy of 0.93. To assess the contribution of individual genes, we utilized the importance() function. We ultimately identified a signature of 24 genes based on a MeanDecreaseAccuracy threshold of > 1. Subsequent clustering analysis using the expression profiles of these 24 genes demonstrated concordance with our prior findings.

**Statistical Methods**

Kendall correlation analysis was performed between microbial KO and host genes. Overall differences in host gene and KO expression among PCA, CA1, and CA2 groups were analyzed using ANOVA, and comparisons between two groups of genes or KOs were conducted using the Student’s *t*-test or Wilcoxon ranksum test. Differences in microbial species and KO expression between CA1 and CA2 groups were analyzed using the Wilcoxon rank-sum test, with standard mean differences calculated as (mean_values “1” - mean_values “2”) / sd (gene_expression), marking gene significance based on *p* value (< 0.05) and mean difference (> log_2_(3/2)). The association between different classifications was evaluated using Fisher's exact test.

* indicates *p* value < 0.05, ** indicates *p* < 0.01, *** indicates *p* < 0.001.

**Supplementary Figures**

**Figure S1.** **Integrated analysis of transcriptome and microbiome profiles in colorectal cancer.** (A) Volcano plot displaying differentially expressed genes between tumor and normal tissues. Red and blue dots represent significantly upregulated and downregulated genes in tumor tissues, respectively (|log_2_FC| > 1, FDR < 0.05). (B) Gene Set Enrichment Analysis (GSEA) of gene ontology (GO) categories for transcriptomic data. The GO categories were enriched in tumor (blue) or normal (orange) tissues. The IL-17 signaling pathway was highlighted due to its association with the immune system. (C) Enrichment score (ES) curve of the IL-17 signaling pathway in GSEA. The y-axis represents ES and the x-axis are genes (vertical black lines) represented in gene sets. (D) Shannon index comparing microbial diversity between tumor (CA) and adjacent normal tissues (PCA). Box plots show median, quartiles, and whiskers; *p* value determined by Student's *t*-test. (E) Simpson index comparing microbial diversity between CA and PCA. Box plots show median, quartiles, and whiskers; *p* value determined by Student's *t*-test. (F) Principal coordinates analysis (PCoA) of bacterial beta-diversity based on Bray-Curtis dissimilarity, demonstrating distinct microbial community structures between tumor (red) and normal tissues (blue).

**Figure S2. Comparison between clusters from transcriptome and modules from microbiome-transcriptome network.** (A) 15 clusters were generated by WGCNA analysis on the transcriptomic data. (B) 31 of 109 genes from M95 overlap with cluster1, 18 of 24 genes generated from random forest overlap with cluster1.

**Figure S3-1. The microbial KO pathway is enriched in each module of the CA group.** (A) Signaling pathways are enriched by KEGG enrichment from M10. (B) Signaling pathways are enriched by KEGG enrichment from M5. (C) Signaling pathways are enriched by KEGG enrichment from M60. (D) Signaling pathways are enriched by KEGG enrichment from M94. (E) Signaling pathways are enriched by KEGG enrichment from M194. (F) Signaling pathways are enriched by KEGG enrichment from M29. (G) Signaling pathways are enriched by KEGG enrichment from M7.

**Figure S3-2. The host gene pathway is enriched in each module of the CA group.** (A) Host gene pathway enriched in module M5. (B) Host gene pathway enriched in module M194. (C) Host gene pathway enriched in module M29. (D) Host gene pathway enriched in module M7.

**Figure S4. M95-based molecular classification.** Unsupervised hierarchical clustering based on M95 gene expression data from both tumor (CA) and adjacent normal tissues (PCA) samples. CA1 samples (light orange) cluster closer to PCA samples (blue) compared to CA2 samples (orange), suggesting molecular similarities.

**Figure S5. Immune signatures of subtypes.** (A) Box plot showing expression patterns of IFNG across PCA, CA1 and CA2 samples. **p* < 0.05, ***p* < 0.01, ****p* < 0.001 by Wilcoxon rank-sum test. (B) Differential expression of interferon-stimulated genes (ISGs) between subtypes. The heat map shows a significantly elevated expression of key ISGs in CA1 compared to CA2 samples. (C) Heatmap showing expression pattern of immune checkpoint genes across CA1 and CA2. (D) Distribution of immune checkpoint GSVA score.

**Figure S6.** **Microbial enrichment of subtypes.** (A) Linear discriminant analysis Effect Size (LEfSe) analysis showing differentially abundant bacterial species between CA1 and CA2 subtypes. Bar length represents log_10_LDA score. Light orange and orange bars indicate species enriched in CA1 and CA2, respectively. (B) Volcano plot displaying differential abundances of bacterial species between CA1 and CA2 samples. Light-orange dots indicate significantly enriched species in CA1, dark-orange dots in CA2 (mean difference > log_2_(3/2), *p* < 0.05).

**Figure S7. Microbial enrichment of subtypes.** (A) LEfSe analysis shows microbial KOs enriched in CA1 and CA2 groups. (B) Wilcoxon rank-sum test shows microbial KOs in CA1 and CA2 groups, similar to LEfSe analysis results. (C) KEGG enrichment of microbial KOs enriched in CA1 from LEfSe analysis, showing butyrate metabolism pathways are enriched in CA1.

**Figure S8. Microorganisms screened from KOs of M95 and IL-17 are similar to CA1.** (A) LOSO analysis shows the top 10 microorganisms contributing most to the M95 module. (B) LOSO analysis of IL-17 signaling pathway-related microbial KOs in the CA group yields the top 10 contributing microorganisms.

**Figure S9. GSEA gene expression difference analysis.** (A) Gene expression enrichment in the two groups of cancer tissues with and without *Bacteroides fragilis*. (B) Gene expression enrichment in the two groups of cancer tissues with and without *Peptostreptococcus stomatis*. (C) Gene expression enrichment in the two groups of cancer tissues with and without *Porphyromonas gingivalis*.

**Figure S10. Clinical validation of CA subtypes.** (A) Heatmap shows expression patterns of identified biomarkers across TCGA samples, with samples classified as CA1-like or CA2-like based on the random forest model. Color scale represents z-scored expression values. (B) Application of the CA1/CA2 classification model to TCGA colorectal cancer cohort (n = 592). Comparison between CA1/CA2 classification and established immune subtypes. (C) Analysis of the composition of CA1 and CA2 of six immune subtypes (ISs), shows that CA1 is mainly composed of type c2, and CA2 is mainly composed of type c1. (D) Comparison with CMS typing shows no obvious pattern, *p* = 0.63.

**REFERENCES**

1.	Nachamkin, I., N. J. Panaro, M. Li, H. Ung, P. K. Yuen, L. J. Kricka, P. Wilding. 2001. "Agilent 2100 bioanalyzer for restriction fragment length polymorphism analysis of the Campylobacter jejuni flagellin gene". *Journal of Clinical Microbiology* 39:754−757. <https://doi.org/10.1128/JCM.39.2.754-757.2001>.

2.	Bolger, Anthony M., Marc Lohse, Bjoern Usadel. 2014. "Trimmomatic: a flexible trimmer for Illumina sequence data". *Bioinformatics* 30:2114−2120. <https://doi.org/10.1093/bioinformatics/btu170>.

3.	Dobin, Alexander, Carrie A. Davis, Felix Schlesinger, Jorg Drenkow, Chris Zaleski, Sonali Jha, Philippe Batut, et al. 2012. "STAR: ultrafast universal RNA-seq aligner". *Bioinformatics* 29:15−21. <https://doi.org/10.1093/bioinformatics/bts635>.

4.	Robinson, Mark D., Davis J. McCarthy, Gordon K. Smyth. 2009. "edgeR: a Bioconductor package for differential expression analysis of digital gene expression data". *Bioinformatics* 26:139−140. <https://doi.org/10.1093/bioinformatics/btp616>.

5.	Sun, Zheng, Shi Huang, Pengfei Zhu, Lam Tzehau, Helen Zhao, Jia Lv, Rongchao Zhang, et al. 2022. "Species-resolved sequencing of low-biomass or degraded microbiomes using 2bRAD-M". *Genome Biology* 23:36. <https://doi.org/10.1186/s13059-021-02576-9>.

6.	Dixon, Philip. 2003. "VEGAN, a package of R functions for community ecology". *Journal of Vegetation Science* 14:927−930. <https://doi.org/10.1111/j.1654-1103.2003.tb02228.x>

7.	Bastian, Mathieu, Sebastien Heymann, Mathieu Jacomy. 2009. "Gephi: An Open Source Software for Exploring and Manipulating Networks". *Proceedings of the International AAAI Conference on Web and Social Media* 3(1):361−362. <https://doi.org/10.1609/icwsm.v3i1.13937>.

8.	Yu, Guangchuang, Li Gen Wang, Yanyan Han, Qing Yu He. 2012. "clusterProfiler: an R package for comparing biological themes among gene clusters". *Omics-a Journal of Integrative Biology* 16:284−287. <https://doi.org/10.1089/omi.2011.0118>.

9.	Liao, Yuxing, Jing Wang, Eric J Jaehnig, Zhiao Shi, Bing Zhang. 2019. "WebGestalt 2019: gene set analysis toolkit with revamped UIs and APIs". *Nucleic Acids Research* 47:W199−W205. <https://doi.org/10.1093/nar/gkz401>.

10.	Zeng, Dongqiang, Zilan Ye, Rongfang Shen, Guangchuang Yu, Jiani Wu, Yi Xiong, Rui Zhou, et al. 2021. "IOBR: Multi-Omics Immuno-Oncology Biological Research to Decode Tumor Microenvironment and Signatures". *Frontiers in Immunology* Volume 12 - 2021. <https://doi.org/10.3389/fimmu.2021.687975>.

11.	Hänzelmann, Sonja, Robert Castelo, Justin Guinney. 2013. "GSVA: gene set variation analysis for microarray and RNA-Seq data". *BMC Bioinformatics* 14:7. <https://doi.org/10.1186/1471-2105-14-7>.

12.	Tang, Zefang, Boxi Kang, Chenwei Li, Tianxiang Chen, Zemin Zhang. 2019. "GEPIA2: an enhanced web server for large-scale expression profiling and interactive analysis". *Nucleic Acids Research* 47:W556−W560. <https://doi.org/10.1093/nar/gkz430>.

13.	Segata, Nicola, Jacques Izard, Levi Waldron, Dirk Gevers, Larisa Miropolsky, Wendy S. Garrett, Curtis Huttenhower. 2011. "Metagenomic biomarker discovery and explanation". *Genome Biology* 12:R60. <https://doi.org/10.1186/gb-2011-12-6-r60>.

14.	Thorsson, Vésteinn, David L. Gibbs, Scott D. Brown, Denise Wolf, Dante S. Bortone, Tai-Hsien Ou Yang, Eduard Porta-Pardo, et al. 2018. "The Immune Landscape of Cancer". *Immunity* 48:812−830.e14. <https://doi.org/10.1016/j.immuni.2018.03.023>.

15.	Kuhn, Max. 2008. "Building Predictive Models in R Using the caret Package". *Journal of Statistical Software* 28:1−26. <https://doi.org/10.18637/jss.v028.i05>.
